# A promising approach to scale up health care improvements in low-and middle-income countries: the Wave-Sequence Spread Approach and the concept of the Slice of a System

**DOI:** 10.12688/f1000research.3888.2

**Published:** 2014-06-25

**Authors:** M. Rashad Massoud, Nana Mensah-Abrampah

**Affiliations:** 1 University Research Co., LLC, Bethesda, MD 20814, USA; 2 Institute of Public Health, University of Heidelberg, Heidelberg, D-69120, Germany

## Abstract

There are several examples of successes in improving health care. However, many of these remain limited to the sites at which they were originally developed. There are fewer examples of successful spread of the improvement more widely inside or outside the health systems within which they were developed. This article discusses the wave-sequence approach to spread or scale up, which enables take up of the improvement in a systematic and sequential way, using “spread agents” — people who participated in the original demonstration sites. The paper also discusses the concept of the “slice” of a system which is useful for thinking about spread and considers a phenomenon related to the rate of adoption which we have observed in this wave-sequence approach.

## Introduction

There are several examples of successes in improving health care. However, many of these successes are limited to the sites where they were originally developed in, a phenomenon referred to as “islands of excellence”. There are fewer examples of success that spread to the remainder of the system where the improvement was originally developed. “Spread” (or “scale-up”) is the science of taking a local improvement (e.g. the implementation of an intervention, the redesign of a process or system) that has produced a better result than the previous method, and actively disseminating it across a system ^1^. There are many ways to “spread”, including, but not limited to, natural diffusion, extension agents, emergency mobilization, collaborative improvement, virtual collaborative methods, campaign spread, the wave-sequence approach, and hybrid models ^1– 3^. Notable examples of spread include the 100,000 Lives Campaign of the Institute of Health Care Improvement; the Quality Assurance Project (QAP) in Russia, which was funded by the United States (US) Agency for International Development (USAID); the USAID Health Care Improvement Project (HCI) in Afghanistan ^4^, and the Project Fives Alive! in Ghana ^5^, which was funded by the Bill and Melinda Gates Foundation. This article focuses on only one of these approaches: the wave-sequence spread approach. It also discusses the associated concept of the “slice” of a system and describes a phenomenon related to the rate of adoption that we are seeing in the wave-sequence approach.

## The wave-sequence approach

The wave-sequence approach is a type of spread that focuses on spreading improved care delivery to other parts of the system. An example of health care delivery system which has yielded better results in a systematic, sequential manner, using spread agents—people who participated in the original demonstration sites—is the implementation of the Active Management of the Third Stage of Labor (AMTSL). Demonstration sites might be hospitals and several clinics, and people from those facilities may assist in spreading the intervention to the remaining facilities in a district, (administrative) department, or city. The term “wave” reflects the fact that this method of spread occurs both sequentially and in an increasingly larger section of the same health care system (perhaps the whole system). Wave-sequence spread is used when it is not possible to cover the whole system all at once. For simplicity, this article uses “wave 1” to refer to the demonstration phase and “wave 2” onwards to refer to the subsequent spread phase; however, some experts use “demonstration” for the first phase and follow with wave 1, etc. Also for simplicity the geographic and administrative unit mentioned in this article refers to the aim of achieving coverage at a whole country level, but the wave-sequence approach can be used at any level: facility, community, district, and on up. It has also been used for spread from an initial ward in large hospitals to the remainder of the wards in those hospitals.

**Figure 1.**
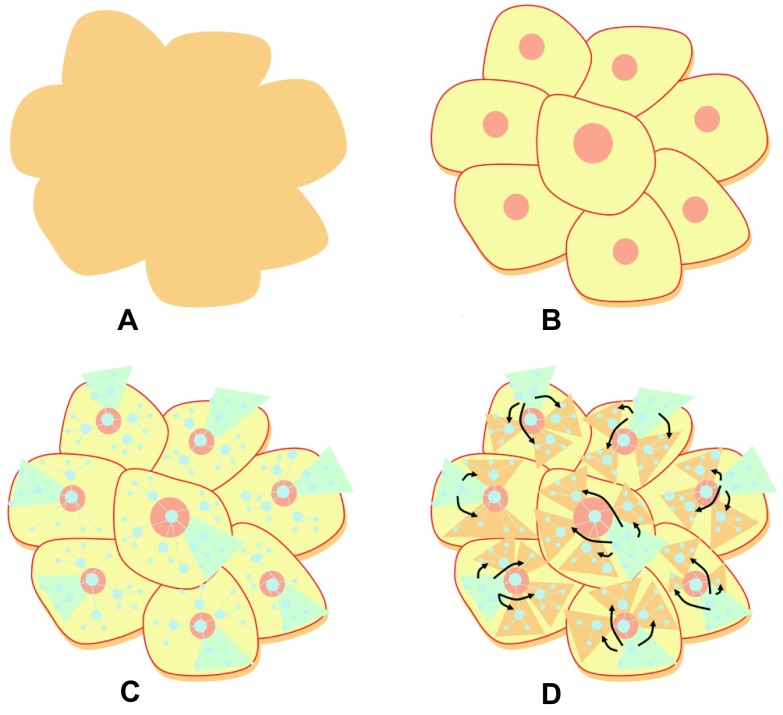
( **A**) Full geographic scale that we want to spread to, covering the entire country. ( **B**) Administrative sub-divisions such as districts, provinces, or states represented as petals in a country. ( **C**) Blue dots representing health facilities and community structures that provide service(s) in a country. ( **D**) Movement of spread agents from their original facilities to facilities in other slices.

The yellow-colored area in Figure 1A represents the full geographic scale we want to spread to, covering the entire country. This would have administrative sub-divisions, such as districts, provinces, or states (this article uses “districts”). Figure 1B presents each of those districts as one of the “petals” of the “flower”. Unlike a real flower, the center of the flower is also referred to as a petal. Each district would have a central city, represented by a red dot.

Throughout the country, health facilities and community structures provide the service(s) for the outcomes we want to improve. These structures are represented by the blue dots in Figure 1C. For the most part in global health, we are dealing with nested systems. These have common administrative structures (usually a Ministry of Health, referral systems, etc.), which are represented by the lines between the blue dots. The green triangles in this figure are described below as “slices” of the system.

The wave-sequence approach is used when we cannot reach the full scale all at once. Therefore, we start with a selection of facilities—those in the wave 1 slices—and later, the staff of the facilities in wave 1 slices will spread the intervention to the remainder of the districts in the system.

## The “Slice of a System” concept

It is best to select a slice of the system in each of the system’s geographic or administrative sub-divisions. A slice (green triangles in Figure 1C) is the set of facilities and community structures (e.g., a community health worker who may work from home) that provide the service that we want to improve and cover a subset of population in each sub-division. The slice has a number of elements ranging from community-level structures to primary care facilities and hospitals including referral facilities. All the structures in a slice would include all the services affecting the outcome of interest (e.g., reducing maternal mortality). This is because patients receive care along the whole continuum of delivery. We would include all those facilities and community structures in the slice to start with. If this were not possible, we would take enough facilities to represent all the different types of facilities in the slice. We work on the initial improvement in these slices, engaging representatives of care providers and their leaders in developing the improvements we intend to make. If for any reason (financial, political, geographic), we cannot take a slice of the system in each sub-division; we take slices in some of the regions but make the selection to the extent possible such that they represent the different settings in the whole target area.

As these improvements are achieved during wave 1, we watch carefully for the providers who are most engaged in the work and produce the best results in the initial set of slices. Towards the end of wave 1, we ask each such provider whether he/she would like to participate in the spread wave(s). For those who accept, we work with them and equip them to become “spread agents” for the subsequent waves that will address the remainder of the system (their spread from their original facilities to facilities in other slices are represented by black lines in Figure 1D). What we look for in the providers is not just the technical skills and skills in quality improvement, but also the ability to teach and coach others in this work. From the beginning of the project, we also make arrangements with the health authorities so that some of their staff will be permitted to play the role of providers or spread agents in the subsequent waves. Arrangements are also made with the health authorities so that they visibly lead the spread in the subsequent waves. Other issues that need to be addressed in the subsequent waves include integrating the data management, communications, meetings, and events. Managing these logistical issues will foster the spread of the intervention into the regular management of the health system.

## Examples of the wave-sequence approach: Russia and Afghanistan

### Russia

**Figure 2.**
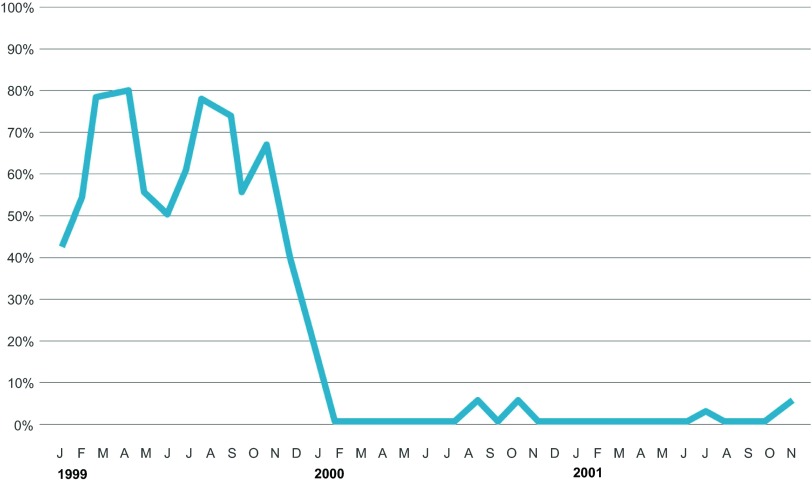
Tver Oblast, Russia: neonates arriving to the neonatal intensive care units (NICU) center with hypothermia. As a result of the neonatal transport system, the percentage of neonates referred to the NICU with abnormally low body temperature was significantly reduced. This improves the survival of newborns with respiratory distress syndrome.

**Figure 3.**
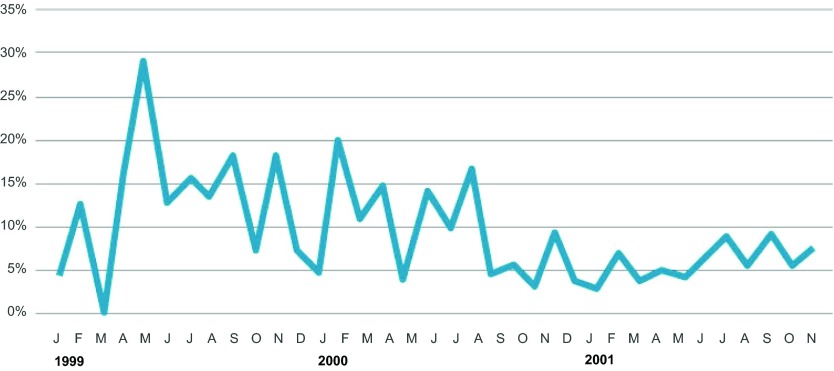
Tver Oblast, Russia: percentages of neonates with respiratory distress who died in the first week of life. As a result of the improvement, fewer neonates died in the first week of life.

Starting in 1998, the USAID QAP worked with their counterparts at the Institute of Public Health in Moscow and the Ministry of Health in Russia on improving care in selected demonstration sites in two of Russia’s 89 oblasts and territories: Tula and Tver Oblasts. The technical areas covered were hypertension (HT), pregnancy-induced hypertension (PIH) and neonatal respiratory distress syndrome (NRDS)—within a couple of years, significant improvements in the quality of care were achieved in all three technical areas. For example, case fatality from NRDS fell by 64% ( Figure 2 and Figure 3). The key changes made to the NRDS care delivery system were to re-organize it into four components:

1. Improve competencies in neonatal resuscitation for pediatricians, obstetricians, midwives and nurses at the point of delivery of newborns;
2. Provide a neonatal transport system consisting of four equipped vehicles;
3. Strengthening the neonatal intensive care units (NICUs) in Tver City, which already existed and were equipped with neonatal ventilation capability. These units received neonates suffering from respiratory distress (a condition requiring referral from the other sites we were working in) from all over Tver Oblast;
4. Implementing policy level changes that facilitated the referral of neonates to higher-level facilities.

The work in Russia used the collaborative improvement method as part of the wave-sequence approach. Collaborative improvement brings together different teams working on a common aim and associated indicators to change and improve processes of care delivery ^3^. Each team collects data on a common set of core indicators to measure whether changes introduced to the system are resulting in improvement. Those changes resulting in improved outcomes are then spread to all the sites involved in the collaborative approach. In itself, collaborative improvement is regarded as a spread approach because it involves a large number of sites working together to improve care. When providers who excelled from the original sites using collaborative improvement become the spread agents to the remainder of the system, this is the wave-sequence spread approach.

After witnessing significant improvements in a relatively short time and the motivating effect that applying improvement methods had on the health providers, the participants in the wave 1 sites turned their attention to how these successes and the use of the improvement methods could be spread further. The goal (set jointly by the USAID QAP, Institute of Public Health in Moscow, Ministry of Health in Russia, and Tula and Tver Oblasts Health Department) for scale up was set from five to 43 hospitals for NRDS, from three to 40 hospitals for PIH (all Tver Oblast), and from five to 442 polyclinics for HT in Tula Oblast ^6^.

The project ultimately had two waves in Tver Oblast and several overlapping waves in Tula, which involved a larger number of facilities. Additional financial resources to support the spread to all of Tula and Tver Oblasts did not come through to QAP despite all good intentions. However, the leadership of both oblasts decided to pursue spread by adding their own resources to QAP’s in order to support this sizeable effort. This culminated in a re-planning of the spread effort with a new design element: The providers who excelled in wave 1 would serve as spread agents to the reminder of their oblast. To maximize their likelihood of success, QAP and the leadership of Tula and Tver Oblasts built their capacity as spread agents. The spread strategy was designed around six concepts ^7^:

1. The spread agents would come from within the system, not from outside. Specifically, members of the quality improvement teams from wave 1 would act as spread agents in the spread waves.
2. Roles and responsibilities were clearly defined. Each spread team had at least one person with strong skills in improvement and another with strong skills in the technical content (NRDS, PIH, or HT).
3. Spread teams were equipped with the competencies needed to effectively perform their duties as spread agents. They were trained in technical content, improvement methods and coaching techniques, so they had not only the know-how, but also could teach and coach others in it.
4. In order to have a mechanism where different spread teams could share experiences and develop hypotheses, spread agents and their teams would join the regular learning sessions that were part of the collaborative improvement method. These sessions are held every six to eight weeks during a collaborative improvement. Representatives of all the facilities participating in that improvement effort attend these sessions ^3^; they offer participants opportunities to discuss experiences, air problems and harvest solutions, and make plans for further improvement efforts when they return to their facilities ^7^.
5. The Oblast Health Authority visibly led the spread effort. Its monthly meetings (“Kollegia” Meetings) became the channel for managing, reporting, communication and problem solving for the spread effort.
6. External technical assistance was provided primarily to the health authorities and the spread agents. The QAP technical assistance team met regularly with the Oblast Health Authority to review the status of the program and to problem solve. A special series of meetings, the “Masters Seminar”, was held in order to enhance the capacity of the spread agents and share learning between them on an on-going basis.

**Figure 4.**
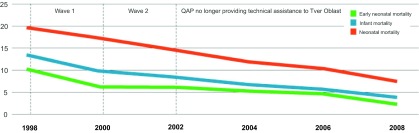
Tver, Russia: improvement of neonatal outcomes. The reduction in early neonatal mortality, the neonatal mortality and the infant mortality during the demonstration phase, spread phase and for six years after the end of USAID QAP’s technical assistance to Tver Oblast are shown. Significant improvements in mortality were achieved and sustained beyond the life of the project. This data comes from Tver Oblast Health Authority surveillance system.

Figure 4 shows the reduction in early neonatal mortality, neonatal mortality and infant mortality during the demonstration phase, spread phase and for six years after the end of USAID QAP’s technical assistance to Tver Oblast. Significant improvements in mortality were achieved and sustained beyond the life of the project. This data comes from Tver Oblast Health Authority surveillance system. The improvement and scale up method used targeted enhancing the capabilities of the staff in Tver Oblast in continually improving care.

### Afghanistan

In 2009, the Afghanistan Ministry of Public Health (MoPH) asked HCI (the follow-on to QAP) to institutionalize improvement methods in Afghanistan. Reducing maternal and newborn mortality and morbidity was chosen as the improvement priority. The improvement initiative initially took place in two provinces, Balkh and Kunduz, together with a referral hospital in Kabul city. The decision to scale up from two provinces and one hospital in wave 1 to four provinces in wave 2 was part of the initial project design. Slices of the system were selected in each of the three sub-divisions and consisted of 26 facilities: 10 facilities in Balkh, 15 in Kunduz, and the referral hospital in Kabul. These 26 facilities constituted wave 1.

Throughout this wave, the collaborative improvement approach was used. Three types of collaborative improvement team were formed:

1. Provincial facility-based collaborative teams,
2. Provincial community collaborative teams, and
3. Kabul Maternity Hospital collaborative teams.

With the help of provincial facility-based and community collaborative teams, HCI prioritized a package of high-impact interventions that was introduced to the selected health facilities. The first phase package focused only on antenatal care, the active management of the third stage of labor, essential newborn care, and immediate post-partum care for the mother. The second phase package addressed antenatal screening for complications, management of obstetric and newborn complications (including eclampsia, sepsis, and newborn asphyxia), and post-partum family planning ^9^. These high-impact interventions were known as the change package.

During wave 2, facility-based quality improvement teams were established at each participating health facility and trained in quality improvement methodology and how sites achieved improvements during wave 1. The providers who excelled in first wave did this. The second wave followed the same pattern, drawing on the change package of evidence-based practices that had been developed from the health facilities.

In Afghanistan, three waves were conducted. Wave 2 focused on spread within the provinces. Once the change package from wave 1 was finalized, the aim was to engage three additional health facilities in each learning session. However, demand for rapid spread was intense yet funding was limited. Consequently, six facilities were added in each wave. Wave 3 focused on spread to other provinces. Once wave 1 was seen to attain a remarkable level of results, spreading the program to other provinces became a priority. In this case, HCI staff served as change agents using the title “provincial coordinator” (PC). PCs were trained and deployed to the original demonstration sites and then became the spread agents for the new provinces. The change package from wave 1 was adapted to fit the needs of the new facilities in wave 2. Spread between major hospitals was part of wave 3. Initially, the PCs started with two major hospitals. After a year, three major provincial hospitals were added. In the second year, five provincial hospitals were added. In total, 11 major hospitals participated in the hospital collaborative improvement.

Scale-up was designed around using local resources and capacity in Afghanistan. Leadership came from within the MoPH of Afghanistan. HCI used local capacity to develop provincial quality improvement teams who developed a context-specific change package, led the improvement, and introduced the successes from wave 1 to the second and third waves. Collaborative improvement approaches and learning prompted the rapid dissemination of successful practices to achieve results.

By the end of 2012, the maternal and newborn health facility collaborative improvement interventions had reached 85 health facilities in the nine HCI-targeted provinces, achieving measurable gains in the quality of maternal and newborn care for an estimated total catchment population of over 1.5 million, about 24% of the population of these provinces.

An important difference between the Russian and Afghan examples is that in Russia, the providers who excelled from the wave 1 site were health providers who led the spread to other sites in the subsequent waves. In Afghanistan, due to local circumstances, it was not possible to use the providers who excelled from wave 1 as spread agents. Instead, HCI staff who were host-country nationals and seen as peers by health staff, worked with the wave 1 sites and undertook the role of spreading the wave 1 experience to the spread sites. In both cases, the common factors were intricate knowledge of the work in wave 1 and being a host-country national with knowledge of the local context.

**Figure 5.**
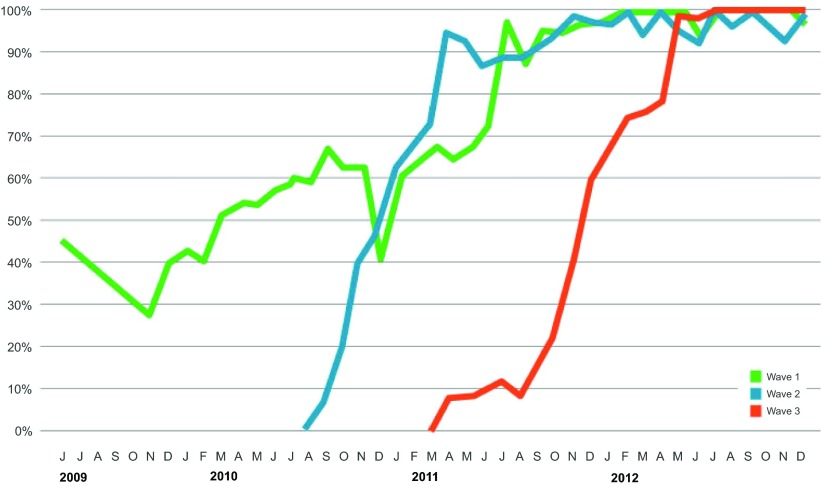
Afghanistan: compliance with Active Management of Third Stage of Labor (AMTSL). The rate of adoption of AMTSL in waves 2 and 3 was significantly faster than in the initial wave.

Figure 5 is a time-series chart from Afghanistan that presents the results of the collaborative improvement that sought to reduce maternal deaths by introducing active management of the third stage of labor. This practice has three services, all delivered immediately after birth to prevent hemorrhage: provision of oxytocin, cord traction, and uterine massage. The graph covers the period June 2009–December 2012, with the results of wave 1 shown in green, wave 2 in blue, and wave 3 in red. Note that the lines for waves 2 and 3 are much steeper than that for wave 1, indicating that the rate of provider adoption of this intervention was much faster than wave 1, a phenomenon that is discussed next.

## Rate of adoption in subsequent waves

It is commonly believed that it is difficult to replicate the results of a well-conducted demonstration or pilot project at a much larger scale and still achieve similarly good results. The experience of many global health care projects has been that as they are scaled up, fidelity to the intervention and its results were not good as in the initial demonstration. This conversation emerged at the end of the scale-up planning session in Russia. Stakeholders agreed “if we can get half as good results in the scale-up as we did in the demonstration, we would be happy”.

Surprisingly though, provider adoption happened faster and more easily and achieved even better results. Implementation of the second and subsequent waves also had fewer problems: spot checks confirmed that the adoption was happening with fewer problems. The ease with which the changes could be transferred and applied by new facilities during subsequent waves can possibly be attributed to the “homophily” factor: the degree to which pairs of individuals who interact are similar in certain attributes, such as beliefs, education, social status and the like ^2^. The spread was conducted by peer providers who had implemented the changes and obtained the improved results in similar facilities in the same context.

**Figure 6.**
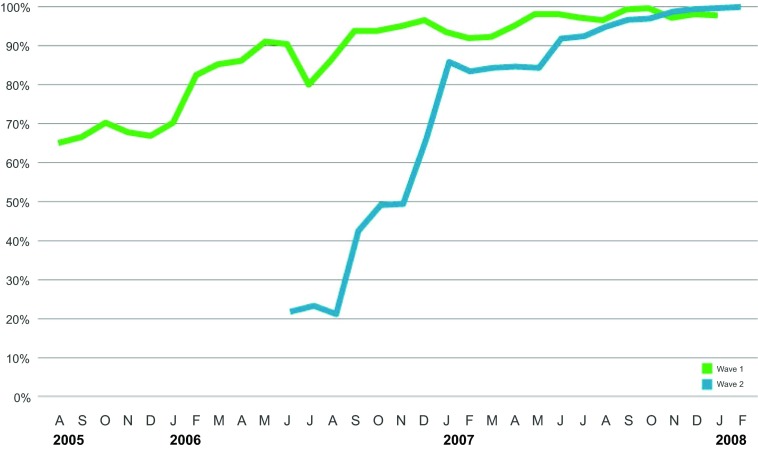
Uganda: compliance with criteria for screening for active TB in HIV-positive patients. The rate of adoption of screening for TB in HIV patients was much faster in wave 2 than wave 1.

We have since seen this faster/easier/better phenomenon in other places and other interventions including AMTSL in Ecuador ^10^, tuberculosis (TB) and HIV in Uganda, Prenatal Care in Guatemala and Integrated Management of Childhood Illness in Niger ( Figure 6– Figure 9). Due to the similarity in context from wave 1 to subsequent waves in these countries, relatively few adaptations were made to the change package developed in wave 1. This rate of adoption phenomenon warrants further research ^11^. Please note that these graphs were chosen to reflect the rate of spread/acceptance/adoption we are seeing with the wave sequence spread approach.

**Figure 7.**
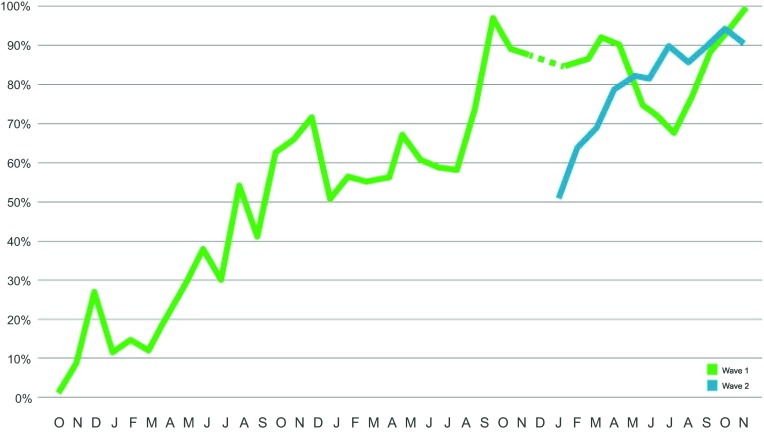
Niger: compliance with criteria for triage of children as part of the Integrated Management of Childhood Illness (IMCI). The rate of adoption of triage as part of IMCI was much higher in wave 2 than wave 1.

**Figure 8.**
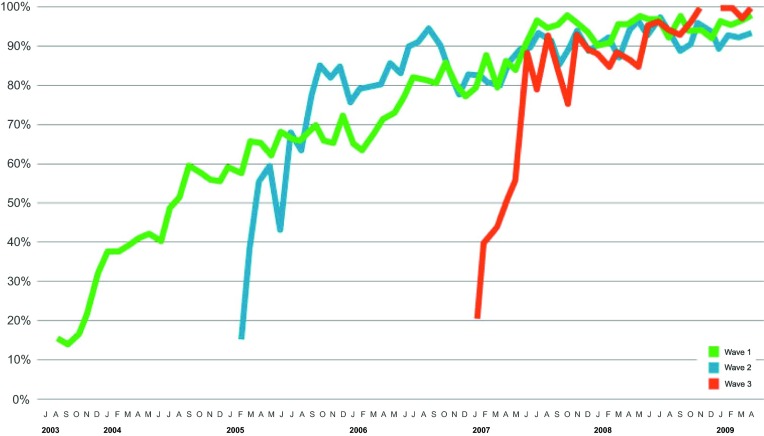
Ecuador: compliance with Active Management of the Third Stage of Labor (AMTSL). The rate of adoption of compliance with AMTSL was much faster in wave 2 and 3 than in wave 1.

**Figure 9.**
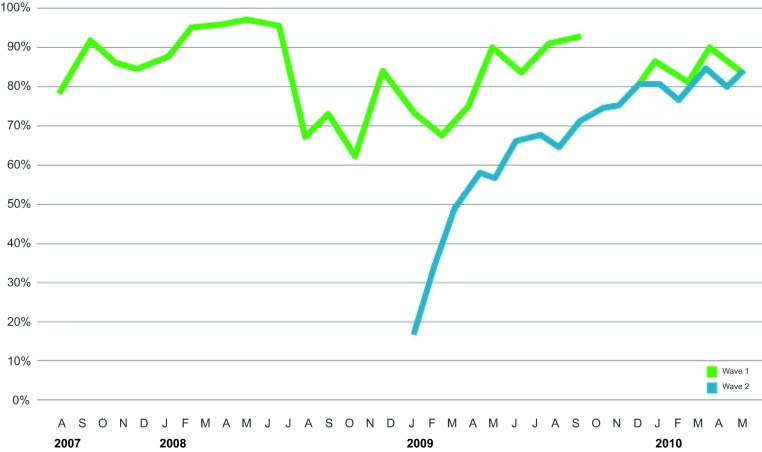
Guatemala: compliance with Prenatal Care Criteria. The rate of adoption of compliance with Prenatal Care Criteria in wave 2 was much faster than in wave 1.

The first graph in Figure 6 shows HCI experience in Uganda with regard to an improvement effort to screen people who were HIV-positive for TB. While both waves reached an 80% adoption [spread/acceptance] rate, wave 2 started at a far lower percentage (albeit with fewer clients) and climbed very steeply compared to wave 1. The second graph shows HCI experience in Niger relative to the adoption of the integrated management of childhood illness. While the wave 1 line seems to stagger up the chart, that for wave 2 rises expeditiously. Again, in Ecuador, the graph shows that waves 2 and 3 moved much more quickly than wave 1, and wave 3 was faster that wave 2. Finally, in Guatemala’s efforts to improve pre-natal care, the wave 1 sites fell from a starting point of almost 80% to a low of 63% before rising to just above 90%. Wave 2, however, made a more rapid rise from 18% to 84%.

## Conclusions

In situations where full-scale adoption of an innovation cannot be reached all at once, the wave-sequence approach has proven to be useful. We believe the reason why an innovation can spread from one set of sites to others is due to the fact that once something has proved to be successful in one set of sites, providers in the following set of sites are more likely to believe it will succeed in theirs. While it has been common for pilot projects to fizzle out when spread to other sites, we believe the successes of the wave sequence approach are based on engaging the host country national staff who have experienced the demonstration wave to champion the spread of implementing the specific changes to others. Testing on a small scale, in the first wave also makes it easier to overcome any managerial, policy-level or other challenges.

It is critical to get results in wave 1 before initiating new waves. Such results give credibility to the proposed changes, giving implementers confidence in the change package. Another important aspect is that though wave 1 can rely on an external development agency, subsequent waves should be much more integrated into the existing national structure of the host-country.
