# Supplementary material for: A promising approach to scale up health care improvements in low-and middle-income countries: the Wave-Sequence Spread Approach and the concept of the Slice of a System
Source: F1000Res. 2014 Jun 25;3:100. Originally published 2014 May 2. [Version 2] doi: 10.12688/f1000research.3888.2 (PMC4184307; doi:10.12688/f1000research.3888.2)
Supplement: Peer review history

# Peer review: Referee response for version 2

**DOI:** 10.5256/f1000research.4906.r5275

**Published:** 2014-06-30

**Authors:** Marie Schall

**Affiliations:** 1 Institute for Healthcare Improvement, Brooklyn, NY, USA

This article provides important detail about the application of the wave approach to spreading improvements and makes an important contribution to the science of achieving results at scale. I have several specific observations and suggestions for ways to strengthen the paper:

**Abstract:**

I suggest that the abstract briefly describes the “phenomenon” that you refer to in the last sentence….I believe this refers to the accelerated pace of the adoption of the changes in each successive wave.

**Introduction:**

The definition of spread given in the introduction refers to spread and “scale-up” as being the same concept.  Is there a rationale for using one term rather than the other?  For most of the paper the term spread is used but there are places later in the paper where the term “scale-up” is used instead.  I’d suggest keeping the language consistent throughout the paper unless there is a reason to use one term versus the other….and to explain that rationale.

**The wave-sequence approach section:**

The first sentence in this section needs to reflect a more precise definition of the wave-sequence approach.  Describing it as a “type of spread that focuses on spreading improved care delivery to other parts of the system” can be used to describe other spread approaches as well.  Introducing the essential components of the wave-sequence approach in the opening sentence would help to distinguish it from the other approaches described in the introduction.  

**The “Slice of a System” concept:**

It would be helpful to give more detail about the composition of the teams in the “wave” and what position/affiliation do the members who become change agents hold. For example, if the selected change agent is a point-of-care provider how do they continue in their provider role while at the same time functioning as a “change agent.”  It would be helpful to give more detail about how the team reflects the infrastructural components of the slice.

**Russia:**

The outcomes of the work are described briefly in the opening paragraph.  Has this work been published elsewhere?  A more precise description of the results in the text is needed, perhaps referring to the timeframe in the graph.  I’d suggest replacing the words “within a couple of years” with the exact timeframe.  

I suggest giving more detail about how the wave-sequence approach was applied in Russia.  For example, what exactly were the petals, what type of facilities made up the slices, and what was the composition of the collaborative teams in each wave?  This detail would help the reader understand the general description of the wave-sequence approach given in the earlier description of the wave-sequence approach.

**Afghanistan:**

A more detailed description of what exactly the “slice of the system” was in this example would also help to explain the general approach (as noted above).

Were the different types of collaborative teams, i.e., provincial facility, provincial community and hospital teams, described for Afghanistan also used in Russia?  Were collaborative teams that involved representatives from each layer in the slice ever used? Did the redesign coordinate effort across these teams? If so, how was that coordination accomplished?

The results for Afghanistan are at the process level, i.e., compliance with the protocols.  Are their outcome levels results? The Russia example showed the actual impact of the changes on neonatal outcomes.  This type of data would strengthen the case example.  

**Conclusion:**

In a subsequent paper I would be interested to hear more about the “managerial and policy level changes” that were made. A greater understanding of these infrastructure changes often plays a key role in the success of large-scale improvement efforts.

I have read this submission. I believe that I have an appropriate level of expertise to confirm that it is of an acceptable scientific standard.

# Peer review: Referee response for version 1

**DOI:** 10.5256/f1000research.4164.r5122

**Published:** 2014-06-16

**Authors:** Lisa Schilling

**Affiliations:** 1 Center for Research in Implementation Science and Prevention, University of Colorado, Aurora, CO, USA

This article appropriately addresses both the social system needs for spread using "spread agents" and a core technical need around identifying the common unit or slice that is involved in adoption and spread. This is important information for healthcare professionals to  understand.  It also explains why a "wave" approach to spread could enhance adoption over the pure diffusion method.

The article does not address the "characteristics" of the intervention itself, complexity for example, nor how adaptation to sites that are somewhat different affects the success of spread. I look forward to subsequent publications that may address such topics.

I have read this submission. I believe that I have an appropriate level of expertise to confirm that it is of an acceptable scientific standard.

# Response

**Published:** 2014-06-17

**Authors:** Nana Mensah-Abrampah

**Affiliations:** USAID Health Care Improvement Project, USA

Dear Lisa,

Thank you for your thoughtful review. We very much appreciate your positive comments. We agree that the article does not address the characteristics of the intervention itself. Given the allowable size of the article, we were not able to discuss several other issues that we would have liked to add. Your idea about subsequent publications that may address this topic is a great one. We look forward to future publications that deal with this.

Thank you,

Nana and Rashad

# Peer review: Referee response for version 1

**DOI:** 10.5256/f1000research.4164.r5011

**Published:** 2014-06-05

**Authors:** John Øvretveit

**Affiliations:** 1 Department of Learning, Informatics, Management and Ethics (LIME), Karolinska Institute, Stockholm, Sweden

- English in abstract – I made slight changes - below is the suggested replacement:
*“* *There are several examples of successes in improving health care. However, many of remain limited to the sites at which they were originally developed. There are fewer examples of successful spread of the improvement more widely in side or outside the health systems within which they were developed. This article discusses a wave-sequence approach to spread or scale up, which enables take up of the improvement in a systematic and sequential way, using “spread agents” —people who participated in the original demonstration sites. The paper also discusses the concept of the “slice” of a system which is useful for thinking about spread and considers a phenomenon related to the rate of adoption which we have observed in this wave-sequence approach.”* 

The title is appropriate for the article, which is well constructed and clear. The methods used as accepted methods for this type of study, and the analyses adequate. The conclusions appear sensible and balanced.

I have read this submission. I believe that I have an appropriate level of expertise to confirm that it is of an acceptable scientific standard.

# Response

**Published:** 2014-06-17

**Authors:** Nana Mensah-Abrampah

**Affiliations:** USAID Health Care Improvement Project, USA

Dear John,

Thank you for your review. We very much appreciate your positive comments. We also appreciate your revisions to the abstract. We agree and have modified the abstract accordingly.

Kind regards,

Nana and Rashad
